# Theater practice and interpersonal synchronization behaviors: a pilot study comparing actors and non-actors

**DOI:** 10.3389/fnhum.2024.1335393

**Published:** 2024-03-11

**Authors:** Gabriele Sofia, Clément Mager, Lionel Brunel, Anne-Sophie Noel

**Affiliations:** ^1^Department of Philosophy, Communication and Performing Arts, Roma Tre University, Rome, Italy; ^2^Epsylon Laboratory EA 4556, Montpellier, France; ^3^UMR5189 Histoire et Sources des Mondes Antiques (HiSoMA), Lyon, France

**Keywords:** theater practice, embodied cognition, performing arts, interpersonal synchronization, entitativity, sense of presence

## Abstract

Recent studies in the field of theater studies no longer view theater as an object, but rather as a dynamic relationship between actors and spectators. In an embodied and situated perspective of cognition, imagination emerges as a product of this dynamic. This study aims to investigate whether acting practice enhances someone’s abilities to set up an effective relationship with others and allows the individual to better manage not only the relationship itself, but also her/his own feelings and those of her/his partner. Eighty two healthy Italian adults, with no communication disorders, including 43 actors (*M*_age_ = 25.4; S.D. = 3.64) and 39 non-actors (*M*_age_ = 24.1 = S.D. = 4.17) completed a joint verbal production task named Random Sequence Generation (RSG) task. Initially, participants performed the task individually. Subsequently, in a second phase, they worked in pairs with another participant, taking turns to contribute to a shared sequence. Pairings were predetermined to ensure a balanced mix of actors and non-actors, and to prevent participants from having prior relationships. Following the task, subjects were queried about their sense of presence, and, their perception of entitativity with their partner. We observed a replication of previous studies, showing higher RSG scores and reduced repetition in the paired condition, indicative of coupling and synchronization behavior. Within pairs, the level of the sense of presence of both partners was positively correlated. Furthermore, an interaction effect between the sense of presence and acting experience on the perception of entitativity was observed. Specifically, actors described perceived entitativity with their partners when their sense of presence was heightened, whereas non-actors experienced a decrease in perceived entitativity with their partners under similar circumstances. We discuss the results and limitations of the study, suggesting the effect of artistic practice on the development of a sort of dual-task ability which enables actors to organize their sensations and actions while sustaining a meaningful connection with others. This research represents an interdisciplinary collaboration between theater studies and cognitive sciences, highlighting the value of a multidisciplinary approach to research.

## 1 Introduction

Since the 1980s, some interdisciplinary fields as “Theater Anthropology” (Barba and Savarese, 2003), “New Theatrology” (De Marinis, 2011), “Ethnoscenology” (Pradier, 2001) and “Performance Studies” (Schechner, 2003) have considered the study of theater no longer as the study of an object, but as the study of the relationship established between actors/actresses^1^ and spectators. In theater, the actor’s task is to create an effective relationship with the spectator. As a matter of fact, the relationship between actors and spectators has grown into a central focus of scientific attention at an interdisciplinary scale: each in our domains (i.e., theater in antiquity, theater studies, and cognitive psychology), we are confronted to the need of developing new hypotheses, models and paradigms to better understand the intensity of the relation established between actors and spectators during a theatrical performance.

We consider the effective relationship as a relationship where the two or more individuals have a feeling of sharing here and now the same event with the other. In this perspective, we want to study the imagination of the actors as one of the means of an effective relationship between actors and spectators. Among all the other theatrical resources mobilized during a show, the imagination is aimed at stimulating the spectators’ attention, which is situated in a specific place and time. In other words, it is a relational and situated imagination.

In the cognitive science field, some researches define imagination as a situated process (Van Dijk and Rietveld, 2020), which emerges from the individual interaction with the environment. From this point of view, in the domain of theater, the spectator’s imagination should emerge from the actor-spectator relationship situated in a specific environment (Sofia, 2013b). Within the limits of this article, we are not directly working on the spectator’s imagination, nevertheless working on the synchronization between actors and non-actors could give us some insight into the actor-spectator relationship which conditions imagination.

We address in our research two concepts specifically used to study the human relation: the feeling of entitativity and the sense of presence.

Entitativity is defined as the degree to which a subject perceives oneself as a part of the same unit bonded together with the other members of a group (Campbell, 1958; Abelson et al., 1998). During an event, the degree of an individual’s immersion into the environment, which depends on the intensity of the coupling with the spatial environment, can be measured by the sense of presence (Usoh et al., 2000; Lee, 2004; Busch et al., 2014).

The sense of presence has been given a range of different definitions in many fields. For instance, many scholars in performance studies have tried to capture the nature of the particular presence possessed by outstanding actors and to theorize the ways in which it can be acquired by students in acting (Barba and Savarese, 2003; Irubetagoyena, 2013; Ruffini, 2014; Taviani, 2022) and it is also investigated in dance (Hansen, 2022; Pini, 2023). For the purpose of this study, we cannot embrace the full spectrum of meaning covered by the sense of presence. We borrow the definition offered by Agrewal et al. (2020) and Lee (2004): we define it as the sense of being fully present in the here and now, intimately connected with both the spatial and temporal environment.

The sense of presence is a key-concept shared by theater studies and cognitive science. From the theater studies point of view, the work of the actor is characterized by a sort of double task: on the one hand, she/he has to manage the relation with the other in order to enhance other’s feeling of entitativity; on the other, she/he has to control and organize her/his own actions and stage behavior in order to enhance her/his own sense of presence (i.e., Barba, 2003).

The fact that theater is an art of relation leads us to suppose that a subject who has a solid experience in theater acting should prove a broader ability in the task of organizing her/his own behavior and in the task of creating a relation with another subject. The effects of her/his theater practice should be observed also during a non-theatrical interaction, when the subject is outside of the theatrical context (Kou et al., 2020).

In order to study how the theater practice modifies the dynamics of relational imagination and interpersonal behaviors, we established an experimental setting based on three research questions:

1. Do people with significant acting experience have better abilities for setting up an effective relationship between them and another individual?
2. Do people with significant acting experience have a greater ability to manage both her/his own feeling of entitativity and sense of presence?
3. Do people with significant acting experience have a greater ability to induce in others (actors or non-actors) an enhanced feeling of entitativity and sense of presence?

In order to study the sense of presence and the feeling of entitativity we focused our experimental research on the interpersonal synchronization behavior. Interpersonal synchronization has been studied through rhythmic, biological, social and motor parameters (Marsh et al., 2009; Lakens, 2010; Lakens and Stel, 2011; Delaherche et al., 2012; Marsh, 2017; Ardizzi et al., 2020). These researchers studied the impact of interpersonal synchronization through movements and rhythmic synchrony on the perception of entitativity. The works by Lakens show that there is a relation between the sensorimotor synchronization (SMS) and the perception of entitativity. Individuals tend to feel more connected to one another when they exhibit similar sensorimotor behaviors and synchronize with the same rhythm (Lakens and Stel, 2011; Czepiel et al., 2021).

During a theatrical performance, physiological synchronization between the audience and the actors has been observed, demonstrating an interactional dynamic and coupling between spectators and actors during a performance (Jola et al., 2012; Sun et al., 2023). Analyzing the sense of presence and the perception of entativity during a non-theatrical interaction between subjects (with or without theatrical experience) allows us to observe how the theater practice modifies the relational abilities of human beings. Therefore, in line with the results found in psychology research on empathy based on performing arts (McDonald et al., 2020), but in adopting a different theoretical perspective and experimental protocole, our study contributes to deepening the understanding of the effect of a theatrical practice on interpersonal relational behavior.

In the field of theater studies, there are some evidence about the effect of theatrical practices on the emotional and social cognition skills (Goldstein et al., 2009; Goldstein and Winner, 2012; McDonald et al., 2020); in the therapeutic contest (Walsh, 2012), i.e., theater with Parkinson’s disease Patients (Mirabella et al., 2017) or theater with children with autism (Beadle-Brown et al., 2018) but there are really few experimental studies about the effect of theater practice involving healthy adult populations. Moreover, there is not any study that explores how the theater practice modifies the relational abilities in a live interaction. In most cases, the experimental setting is mediated by a screen (Calvo-Merino et al., 2008; Dezecache et al., 2013). From that point of view, our experience could be considered as a pilot study toward the analysis of the effect of theater practice in a live interaction.

## 2 The current study

Our study reuses the RSG task described by Towse et al. (2016). The RSG task is a joint task used for the measure of interpersonal number series productions. This task is based on the Random Number Generation task established by Evans (1978) which is a tool of number randomization production measure in attentional research.

In their studies, Towse et al. (2016) have shown that human production is significantly different from computer-generated production. Given that it appears impossible for humans to generate true randomness, individuals will instead employ their own production of algorithms, thereby crafting the illusion of randomness. Thus, in paired productions, individuals seek to modify their numerical production schemas to align with each other and to assimilate their partner’s schema. This process incurs an additional cognitive cost, but also results in a decrease in repetition frequencies (Towse et al., 2016).

Numerous studies have drawn upon Towse’s earlier research, especially in the examination of repetition avoidance mechanisms (Yang and Bao, 2023). Additional investigations by Maehara et al. (2019) concentrated on deciphering the social structures influencing significant joint cognitive phenomena during the RSG task. Their findings suggested that joint cognitive phenomena may primarily stem from turn-taking configuration rather than social dynamics. Also, Hartmann et al. (2019) have shown that individuals in interpersonal production situations share a mental number line rooted in spatial processes. Thus, the spatial position of the co-actor influences the individual’s production, and this phenomenon only emerged during the paired productions.

All of this recent research underscores a specific focus on employing the RSG task to investigate interpersonal interaction behaviors. Although studies have been conducted to investigate the impact of artistic or theatrical practices on individual’s behavior during a joint task (see above), none of them has used Towse’s instrument to do so. In our present study, we have explored whether a theatrical experience influences interpersonal relational behavior, and the feelings of both oneself and one’s partner regarding the experience. To show that, we choose to focus our research on the sense of presence (Usoh et al., 2000; Lee, 2004), and the perception of entitativity (Campbell, 1958; Abelson et al., 1998; Lickel et al., 2000) experienced by the subjects (with or without theatrical background) during the RSG task.

## 3 Method

### 3.1 Participants

Our population comprised 82 Italian adults aged between 18 and 30 (*M*_age_ = 24.8; S.D. = 3.93), including 48 women and 34 men (Table 1). All participants were Italian nationals residing in the metropolitan area of Rome, with a strong upbringing in Italian culture and using Italian as their primary language.

**Table 1:** Headcount (number of women), age (standard deviations) and score of artistic practice (standard deviations) of participants depending on the acting experience.

Subjects	Sample	Age	Artistic practice
Actors	43 (24)	25.4 (3.64)	44.4 **(13.1)
Non-actors	39 (24)	24.1 (4.17)	25.1 **(16.3)

We observed a significant difference on artistic practice scores between actors and non-actors on the student *t* test for independent samples; *p < 0.05; **p < 0.001.

Our population consisted of 43 actors (M_age_ = 25.4; S.D. = 3.64) and 39 non-actors (M_age_ = 24.1 = S.D. = 4.17) divided into 15 pairs of actors (*M*_age_ = 24.8 = S.D. = 3.39; 16 women and 14 men), 13 pairs of non-actors (*M*_age_ = 23.8 = S.D. = 3.48; 15 women and 11 men) and 13 mixed pairs (one actor with one non-actor, *M*_age_ = 25.6 = S.D. = 4.79; 17 women and 9 men). This arrangement ensured that there were interactions between actors and actors, actors and non-actors, non-actors and actors, as well as non-actors and non-actors. Actors were individuals who had engaged in continuous weekly theater practice for a minimum of 3 years, whether amateur or professional, and were still actively practicing at the time of the experiment. Non-actors were participants with no regular theatrical experience at the time of the experiment. The gender distribution of the subjects in the pairs was counterbalanced. The pairs did not know each other before the experiment.

Participants were recruited through advertisements on social networks, as well as from Italian actors’ groups and university theater companies in Rome. Participants had no sensorimotor, social communication or pragmatic disabilities or disorders (see DSM-V). Subjects were volunteers, with no inducements, and signed a consent form of their own accord, fully informed, before commencing their participation. Before taking part in the experiment, all the participants completed artistic practice questionnaires which confirmed a significant difference in theatrical practice between our groups (Table 1). The study was approved by the Ethics Committee of Roma Tre University (Report n. 5, 11 April 2023).

### 3.2 Materials and stimuli

#### 3.2.1 Apparatus

For the RSG task, we created a metronome using OpenSesame 3.3.10 software, which emitted a sound signal (440 Hz) at 1.5 s intervals for a duration of 100 ms. This sound signal was accompanied by a visual cue in the form of a navy-blue arrow consisting of 288 pixels, sequentially directing the participant who needed to generate the item. The software was run on a laptop with Intel^Ⓡ^ Core™ i7-8750H 2.2 GHz, DDR 8GB RAM, built-in speakers and a NVIDIA GeForce GTX 1050 4GB, running Microsoft Windows 10. The screen size was 17.3″ (38 × 21.5 cm) in 1920 × 1,080 full HD resolution. The laptop was placed on a table 70 cm away from the two participants.

Another laptop with a similar composition was used by experimentators to put responses’ production on Excel during the task.

#### 3.2.2 Experimental questionnaires

Immediately after the RSG task, participants were asked about their feelings during the task. First, they were asked about their level of presence (ranging from 7 to 70) during the task (spatiotemporal coupling with the subject’s physical environment) using the SUSPQ questionnaire (Usoh et al., 2000), with the questions tailored to the experimental cubicle as a reference point. The SUSPQ questionnaire consisted of 10 self-assessment items on a 7-point Likert scale, including 2 inverted items. The questionnaire generated a presence score that fell between 7 and 70, with a high score indicating a strong sense of presence within one’s spatiotemporal environment, and conversely, a low score indicating a weaker sense of presence. An illustration of an item from the sense of presence questionnaire is provided below. It has been translated into English, given that the surveyed population was Italian, and the experimental questionnaire was written in Italian. The entire experimental questionnaire is available at the end of this paper.

*Item from our experimental questionnaire (adapted to our experimental situation)*:

Please rate your sense of being present in the experimental room during the task, on the following scale from 1 to 7? I had the feeling of “being there” in the room:

Not at all 1–2 - 3 - 4 - 5 - 6 – 7 All the time.

*Original Item from SUSPQ questionnaire* (Usoh et al., 2000):

Please rate your sense of being in the office space, on the following scale from 1 to 7, where 7 represents your normal experience of being in a place. I had a sense of “being there” in the office space:

Not at all 1–2 - 3 - 4 - 5 - 6 – 7 Very much.

Subsequently, participants were inquired about their level of entitativity (between 16 and 144) within their pair (coupling in the relation with the other) using the EQ questionnaire (Lickel et al., 2000). The Entitativity Questionnaire consists of 16 self-assessed items on 9-point Lickert scales, including 4 inverted items. Participants obtained entitativity scores between 16 and 144, reflecting their perception of the level of entitativity within their pair. An illustration of an item from the entativity questionnaire is provided below.

*Example of item from the entitativity questionnaire*:

To what extent do you consider your partner to be a “whole,” a common entity? I feel that my partner and I formed a common entity during the task:

Not at all 1–2 - 3 - 4 - 5 - 6 - 7 Absolutely.

### 3.3 Procedure

Upon arrival, participants were asked to complete an artistic practice questionnaire and provide their consent by signing a consent form. Following this, the two participants within each pair were gathered in the waiting room, where the experimental procedure was explained.

Subjects were first taken individually, one after the other, to perform the RSG task in individual production of 100 numerical items ranging from 1 to 10 in a random manner, without adhering to any specific logic, and avoiding consecutive repetitions, as much as possible. Participants were instructed to produce one item every 1.5 s, following the rhythm set by the metronome. To ensure comprehension of the task and materials, a training session involving the production of 10 items was conducted before commencing the actual task. This procedure comes from previous experimental research developing the RSG task (Towse et al., 2016).

Next, the pairs were brought together to perform the task in a paired condition. The instructions remain the same as for the individual condition. Participants were required to take turns producing an item every 1.5 s, in synchronization with the metronome. Similarly, a training session involving the joint production method, comprising the production of 10 items, was conducted to ensure that participants understood the process before proceeding to jointly produce 200 items.

Immediately after the task, participants completed the presence questionnaire (Usoh et al., 2000), followed by the entitativity questionnaire (Lickel et al., 2000) in the experimental cubicle. Once the questionnaires had been completed, a discussion took place between the participants and the experimenters to address any queries, clarify the objectives, and gather feedback.

### 3.4 Data analysis

#### 3.4.1 Preprocessing

During the task, each production was logged in real-time by an experimenter using an Excel document. As a result, for each pair, we generated three separate Excel files: one for each individual’s production and one for the pair production.

Subsequently, all of these files were processed using the RGCalc software (Towse and Neil, 1998; Towse et al., 2016) to analyze the numerical series and extract two key indicators: the RNG Score and the R%. The RNG Score (Evans, 1978) is a widely used measure of randomization performance, which assesses the distribution of responses by employing an *a* × *a* matrix to evaluate how frequently one response alternative follows another. The RNG score provides a performance score for the randomization task, ranging from 0 (perfect equality of diagram distribution) to 1 (complete predictability of numerous sequences).

The R% (redundancy frequencies) serves as an indicator of repetition by quantifying the extent of deviation from the ideal information generation (Towse and Neil, 1998). Consequently, an R% of 0% means a state of perfect equality in response alternative frequencies (implying no redundancy), while conversely, an R% of 100% indicates complete redundancy.

All the equations for calculating the RNG score and R% are available and explained in the original article by Towse and Neil (1998).

#### 3.4.2 Method of analysis

In order to test our hypothesis, we ran three distinct analyses:

In the first analysis, we performed a General Linear Mixed Model (GLMM) with acting experience (actor vs. non-actor) and condition (solo vs. duo) as fixed factors, and participants as a random factor. This analysis aimed to assess the interaction between acting experience and the condition in relation to both RNG and R% scores.

In the second analysis, we executed a GLMM with acting experience (actor vs. non-actor) as the fixed factor, the sense of presence score as a covariate, and participant as the random factor, in order to test the interaction between acting experience and presence on entitativity scores.

For the third analysis, we conducted a GLMM with acting experience (actor vs. non-actor) and the type of partner (actor or non-actor) as fixed factors, and either the sense of presence score or the perception of entitativity score as a covariate, with participants as the random factor. This analysis was designed to investigate the impact of acting experience on the partner’s sense of presence and perception of entitativity.

For all our analyses, we used Jamovi (version 2.3.28) and the GAMLj package (reference). During the assumption-checking process, outliers were detected only in the first analysis, and they were subsequently removed (2 RNG scores and 4 R%). We maintained a significance level at 0.05 and conducted tests for simple effects in all significant interactions.

## 4 Results

For sake of clarity, only omnibus ANOVA results were reported here (Full analysis as well as the data are available on OSF https://doi.org/10.17605/OSF.IO/BPT5D; Table 2).

**Table 2:** Descriptive statistics including means and standard deviation of all participants’ RSG scores, *R* %, sense of presence scores and feeling of entitativity scores.

		Condition	RSG score	*R* %	Sense of presence score	Feeling of entitativity score
Actor	Means	Individual	0.307	0.021	45.2	76.1
		Pair	0.350	0.008		
	Standard deviation	Individual	0.045	0.019	7.79	14.7
		Pair	0.019	0.007		
Non-actor	Means	Individual	0.305	0.015	45.7	77.5
		Pair	0.346	0.007		
	Standard deviation	Individual	0.069	0.012	7.83	15.4
		Pair	0.017	0.004		

### 4.1 Individual vs. paired

Analysis on performance revealed a significant effect of condition (individual or pair production) on participants’ RNG score, *F*(1, 158) = 82.982, *p < 0.001*, with a lower RNG score in individual condition (Estimated Marginal Mean = 0.301, SE = 0.003) than in paired condition (Estimated Marginal Mean = 0.348, SE = 0.003). Since a lower RNG score means better randomization of production, it seems that participants produce more random sequences when they were alone than in paired conditions. There was also a significant difference in R% between the two conditions, *F*(1, 152) = 35.212, *p < 0.001*, with higher redundancy (Estimated Marginal Mean = 0.0155, SE = 9.57e^−4^) in individual conditions than in pairs (Estimated Marginal Mean = 0.0074, SE = 9.57e^−4^).

We did not observe any effect of the acting experience (actors vs. non-actors) on RNG scores [*F* (1, 158) = 1.95, *ns*.] and R% [*F* (1, 152) = 2.603, *ns*.]. Likewise, we observed no significant effect of the interaction between the condition and acting experience on randomization performance.

### 4.2 Managing their own feelings

In terms of subjective experience, we did not observe any statistically significant main effect of acting experience on the perception of entitativity: *F*(1, 132) = 0.373, *ns*. Similarly, the sense of presence score, used as covariate, did not appear to have any significant effect on the perception of entitativity among participants: *F* (1, 160) = 0.222, *ns*.

**Figure 1:**
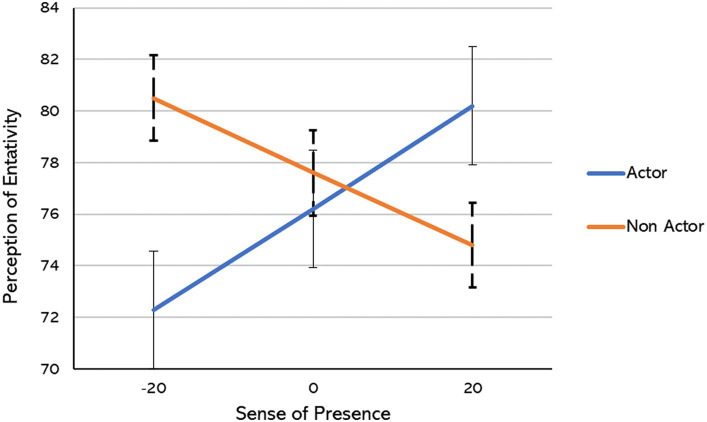
Estimated Marginal Means self-reported perception of entativity score between paired subjects (min = 72.3; max = 80.5), depending on their own sense of presence centered score and their acting experience (actor or non-actor).

We observed a significant interaction effect of sense of presence score and acting experience on perception of entitativity: *F* (1, 160) = 8.712, *p* = 0.004. As depicted in Figure 1, a low sense of presence score among participants is associated with a correspondingly low perception of entitativity scores for actors (Estimated Marginal Mean = 72.3; SE = 2.20). In contrast, the perception of entitativity scores for non-actors remains relatively high (Estimated Marginal Mean = 80.5; SE = 2.39). Conversely, when participants report a high sense of presence score, the perception of entitativity scores appears to be elevated for actors (Estimated Marginal Mean = 80.2; SE = 2.27). In this scenario, the perception of entitativity scores is lower for non-actors (Estimated Marginal Mean = 74.8; SE = 2.30).

Upon analyzing the simple effects of acting experience, it became evident that a significant difference in the perception of entitativity existed when participants reported a low presence score (Mean-1-SD): *F* (1, 150) = 6.336, *p* = 0.013. In contrast, no significant difference was observed at medium and high presence scores. Nevertheless, under conditions of high presence scores (Mean + 1-SD), the effect appears to be marginal [*F*(1, 151) = 2.761, *p* = 0.099], thereby affirming the previously observed trend (refer to Figure 1).

### 4.3 Managing the other feelings

We identified a significant correlation between participants’ sense of presence scores and those of their partners: *F*(1, 157) = 10.323, *p* = 0.002. It appeared that sense of presence scores within pairs exhibited a linear trend: when individuals’ scores increased, their partners’ scores also tended to increase. We did not observe any significant main effect of the individual’s acting experience or the type of partner on the partner’s sense of presence.

A significant effect of the individual’s acting experience on the partner’s perception of entitativity score was observed: *F*(1, 8.541) = 6.955, *p* = 0.028. Partners tended to perceive higher entitativity when the individual was an actor (Estimated Marginal Mean = 79.4; SE = 1.74) compared to when the individual was a non-actor (Estimated Marginal Mean = 72.9; SE = 1.81). We did not observe any significant main effect of the type of partner or individual’s perception of entitativity score on the partner’s perception of entitativity. All interaction effects appeared to be non-significant for partners’ sense of presence and perception of entitativity.

## 5 Discussion

The objective of our study was to understand the effect of a theatrical practice on interpersonal relational behavior. Relation is indeed at the core of theater and the imagination of the spectators is greatly dependent on the art of the actor, and in particular, her/his capacity to be in a state where she/he feels imaginary things as if they were real (see Van Dijk and Rietveld, 2020). To do so, we used a joint RSG task (Towse et al., 2016) as it was already used in the literature to study interpersonal behaviors. In this paradigm, participants are engaged in a random number generation task in a solo condition and then in duo condition. First of all, our results are in line with prior studies using this paradigm: they indeed confirm a modification of the performance in terms of randomness between the solo condition and the duo condition (participants are less random but also do less repetitions in the duo condition compared to the solo condition). Usually, this result is interpreted as evidence that a coupling process is occurs between the two participants during the paired condition.

More specifically, our study aimed at addressing these three research questions:

1. Do people with significant acting experience have better abilities for setting up an effective relationship between them and another individual?

2. Do people with significant acting experience have a greater ability to manage both her/his own feeling of entitativity and sense of presence?

3. Do people with significant acting experience have a greater ability to induce in others (actors or non-actors) an enhanced feeling of entitativity and sense of presence?

To deal with these questions we manipulated the acting experience of our participants so that half of our sample was considered as actors and the other half not. To answer the second and the third question, we measured both the sense of presence (Usoh et al., 2000) and the perception of entitativity (Lickel et al., 2000) for participants after the duo condition. This led to three mains results:

1 - We did not find evidence that people with significant acting experience have better abilities for setting up an effective relation between them and another individual. Indeed, we did not find a significant interaction between acting experience and the condition (solo vs. paired) on randomness scores. The analysis initially reveals no significant impact of theatrical experience on this task, suggesting that theatrical experience may not influence relational skills. However, it’s crucial to discuss the results in light of former literature (i.e., former studies on the specific empathetic skills of actors, McDonald et al., 2020) and also in light of observed effects on other aspects of the relationship (i.e., question 2 & 3).

In the task we chose, the actors are not explicitly required to perform as “actors.” Their abilities are indirectly elicited in a context that does not prompt them to adopt a theatrical posture, such as strong physical engagement (as discussed by Sofia, 2013a). Instead, the task resembles an everyday situation. It is also possible that the measurement may lack sensitivity or statistical power to detect the effect of artistic experience on randomization scores, particularly when this effect is overshadowed by the significant impact of the condition (solo vs. paired). The measure of randomization may not be suited to observe the influence of theatrical practice on relational skills, explaining why we observe an effect on participants’ feelings about the task but not on randomization scores.

In this task, the attention given to the other in the paired condition seems to transcend differences in theatrical experience. This might also be due to some participants of the experience possessing unmeasured synchronization skills, unrelated to theatrical practice, acquired independently in different social contexts. For example, they may be accustomed to working in groups, engaging in collaborative teamwork, or participating in professional or leisure activities where interpersonal relationships play a central role.

We already know that during joint action, the nature of the relation (such as romantic relation, see Quintard et al., 2021; or friendship, see Ford and Aberdein, 2015), the psychological disposition of the participants (personality, Ford and Aberdein, 2015; Walter et al., 2021; Campos-Moinier et al., 2023) and the physical constraints (e.g., position of the participants, Tsai et al., 2006; Hartmann et al., 2019) are factors that impact the performance. So that, for instance, friends or lovers appeared more coupled than strangers. To the best of our knowledge, our study is the first study in the joint literature that manipulates the experience/skill of participants to establish and manage interpersonal relations while controlling the nature of relation (our participants were strangers). Despite joint performance seemed unaffected by this manipulation, we found interesting results showing that this manipulation affects other perceptions about coupling (i.e., entitativity).

2 - People with significant acting experience have a greater ability to manage both their own feeling of entitativity and sense of presence. Their perception of entitativity increases proportionally along with their sense of presence whereas, for participants with less or without acting experience, these are negatively correlated (the higher their sense of entativity is, the weaker their sense of presence). This can be interpreted as proof of better abilities in participants with acting experience in maintaining concomitantly a high level of coupling with others and an attention to her/his own sense of presence in their spatiotemporal environment during the task. This may be related to an ability developed by actors during their performance to stay alert and aware of what is going on around them, while maintaining the relation with the others (who can be spectators in a theatrical situation).

3 - People with significant acting experience have a greater ability to induce a better perception of entitativity in the others (actors or non-actors). Moreover, we found evidence of a correlation between the subject’s sense of presence and that of her/his partner (whether actor or non-actor). Individuals seem to share a higher sense of presence when their partners also experience a high sense of presence.

These results first suggest the actors’ increased abilities to make the others (whether they are themselves actors or not) enter in an efficient coupling with them, through a better perception of the group’s entitativity. Secondly, in a situation of interaction, the fact that someone’s sense of presence is proportional to that of her/his partner highlights the relational nature of it.

Among the three results, the second is the most relevant. It shows how the theater practice modifies both the individual’s entitativity and sense of presence dynamics during an interpersonal interaction. This could be part of the cognitive dynamics of actor-spectator imagination during a theater performance. Already in antiquity, the first-century AD Roman rhetor Quintilian thus noted that when performing, an actor experienced ‘the paradoxical state of feeling imaginary things as if they were real, a state which they are able to induce in their audiences as well’ (Webb, 2009: 104, see *Institutio Oratoria,* 6, 2, 29–30). Therefore it is also interesting to situate our experimental study in a *longue durée* perspective, as one that can enter in dialog with current researches on modern but also historical forms of theater (such as Meineck, 2017; Budelmann and Sluiter, 2023).

For the field of theater studies these results show, first of all, that the acting experience modifies the ability of organizing relationships with others, also in a non-theatrical context. This could partially explain the efficacy of theater practice also when it is not aimed at the production of a performance. In fact, recent studies show that theater practice has a strong impact as complementary therapy in clinical, pedagogical and social integration contexts (Modugno et al., 2010; Mirabella et al., 2017; McDonald et al., 2020).

In particular, our results allow us to bring a positive answer to the second research question we ask: people with significant acting experience indeed seem to have a greater ability to manage both their own feeling of entitativity and sense of presence. In our experiment, only persons with a strong theatrical baggage experienced at the same time a high sense of presence and a high level of entitativity. This could lead us to think that theater practice trains a subject in building up an efficient relationship with others without losing her/his sense of presence in the environment. This could be explained by the fact that the actor must always accomplish onstage a simultaneous double task: on the one hand, she/he has to perform with a high degree of precision the actions contained in the scenic score, on the other, she/he must stimulate and maintain the attention of the spectators (Sofia, 2013b). This forces her/him to develop a different self-organization, a phenomenon which has been described under different names, according to the different theater techniques, i.e., sincere body (Copeau, 1955), decided body (Barba, 2003), biomechanical body (Meyerhold, 1969), extra-daily organization, etc.

This experimental context highlights that the training pursued to acquire a ‘scenic body’ may also modify actors’ social abilities, whether they are on stage or not. The dynamics discovered may arguably be transferable to theatrical or real-life contexts, but further studies would be needed to affirm it more solidly. Within this experiment, we have shown for the first time an effect of acting experience on interpersonal behaviors in a ‘social’ live context (although the lab experiment conducted under specific conditions only partially reflects what happens in daily-life social relationships). Further research could be instrumental in fostering a better understanding of the efficacy of theater practice in therapeutic, pedagogical or social integration contexts. In all these contexts, studies of imagination in an embodied perspective (Gallese and Sinigaglia, 2018) can also be useful inasmuch imagination processes do not only foreground artistic works, but are the basis of all socio-cognitive dynamics, such as social integration, interpersonal synchronization, and improvement of social skills, which also suggests a rich array of potential perspectives for future research.

The present study design and analysis of findings also involved productive interdisciplinary exchange about concepts (presence, entitativity), methods, and the interpretation of results in light of historical and contemporary critical understandings of the actor’s embodied competences and imagination. We hope it can encourage scholars to bridge the gaps between the different disciplines and work toward more and more integrated forms of synergy between experimental quantitative methodology and artistic, critical scholarly research methods, and epistemology, from theater studies.

## 6 Conclusion

In summary, this study has identified an effect of acting experience on the way a task involving interpersonal relationship is approached, as well as on the feelings experienced by the interacting individuals. During an interpersonal synchronization task, which requires entering into a relationship with others, actors seem to better manage their own and the partner’s feelings. This research represents a continuation of the collaborative effort between cognitive science and the study of theater and live performance, (as previously explored by Lutterbie, 2006; Falletti et al., 2016; Lippi et al., 2016; Shaughnessy and Lutterbie, 2016 and, for an historical overview, Sofia, 2014). It underscores the ongoing significance of interdisciplinary collaboration in understanding the human experience. We advocate for the continued sharing of knowledge and expertise concerning both the spectator and the actor. Such a collaboration fosters multidisciplinary research, helping us further unravel the impacts of artistic practice on human behavior. Therefore, our study opens the way for future investigations into relational behaviors. Exploring further the dynamics operating during the task could be enhanced by employing live dynamic measurements in order to observe the mechanisms behind oscillatory coupling.

## Data availability statement

The datasets presented in this study can be found in online repositories. The names of the repository/repositories and accession number(s) can be found at: https://doi.org/10.17605/OSF.IO/BPT5D.

## Ethics statement

The studies involving humans were approved by Ethics Committee of Roma Tre University (Report n. 5, 11 April 2023). The studies were conducted in accordance with the local legislation and institutional requirements. The participants provided their written informed consent to participate in this study.

## Author contributions

GS: Conceptualization, Funding acquisition, Investigation, Methodology, Project administration, Supervision, Writing – original draft, Writing – review & editing. CM: Conceptualization, Investigation, Methodology, Project administration, Software, Supervision, Writing – original draft, Writing – review & editing, Formal analysis. LB: Conceptualization, Formal analysis, Methodology, Writing – review & editing. A-SN: Conceptualization, Writing – review & editing.

## Footnotes

^1^Ideally, we would like to use both masculine and feminine terms (actors and actresses, non-actors and non-actresses) all throughout this article. However, for the sake of greater clarity and legibility, from this sentence on, we will use only the masculine terms “actor” to designate in a generic way both female and male individuals.
