# Supplementary material for: Theater practice and interpersonal synchronization behaviors: a pilot study comparing actors and non-actors
Source: Front Hum Neurosci. 2024 Mar 11;18:1335393. doi: 10.3389/fnhum.2024.1335393 (PMC10961450; doi:10.3389/fnhum.2024.1335393)
Supplement: Supplementary file 1 [file Data_Sheet_1.docx]

# EXPERIMENTAL QUESTIONNAIRES

**CODICE PARTECIPANTE: _______________________** Età: ____________

Hai dei problemi di comunicazione o di autismo? __________

**QUESTIONARIO SULL’ESPERIENZA ARTISTICA E L’ATTIVITÀ FISICA**

**1/** Da quanti anni fai teatro? (Segna 0 se hai mai fatto teatro):

0 – 1 – 2 – 3 – 4 – 5 – 6 – 7 – 8 – 9 – 10

**2/** Durante la pratica teatrale, lavori generalmente da solo/a o in gruppo? Durante la pratica teatrale lavoro generalmente:

Da solo/a 1 – 2 – 3 – 4 – 5 – 6 – 7 In gruppo

**3/** Nel tipo di teatro da te praticato quanto è coinvolto il corpo? Nel tipo di teatro da me praticato il corpo è coinvolto:

Pochissimo 1 – 2 – 3 – 4 – 5 – 6 – 7 Moltissimo

**4/** Nel tipo di teatro da te praticato, quanto è utilizzata la tua voce? Nel tipo di teatro da me praticato la mia voce è utilizzata:

Pochissimo 1 – 2 – 3 – 4 – 5 – 6 – 7 Moltissimo

**5/** Nel tipo di teatro da te praticato, quanto è utilizzato il tuo sguardo? Nel tipo di teatro da me praticato il mio sguardo è utilizzato:

Pochissimo 1 – 2 – 3 – 4 – 5 – 6 – 7 Moltissimo

**6/** Quale spazio dai all’improvvisazione nella tua pratica teatrale? Nella mia pratica teatrale lo spazio dato all’improvvisazione è:

Pochissimo 1 – 2 – 3 – 4 – 5 – 6 – 7 Moltissimo

**7/** Con quale frequenza pratichi dello sport? La frequenza con cui pratico dello sport è:

Mai 1 – 2 – 3 – 4 – 5 – 6 – 7 Ogni giorno

**9/** La tua pratica sportiva è generalmente individuale o collettiva? La pratica sportiva è:

Individuale 1 – 2 – 3 – 4 – 5 – 6 – 7 Collettiva

**9/** Durante la tua vita professionale o i tuoi studi, quanto è coinvolto il tuo corpo? Nella mia vista professionale o nei miei studi il mio corpo è coinvolto:

Pochissimo 1 – 2 – 3 – 4 – 5 – 6 – 7 Moltissimo

**10/** Con quale intensità pratichi danza? Ho una pratica della danza:

Molto scarsa 1 – 2 – 3 – 4 – 5 – 6 – 7 Quotidiana

**11/** Con quale intensità svolgi una pratica somatica (yoga, meditazione, Feldenkrais, arti marziali ecc.)? La frequenza di una pratica somatica è:

Molto scarsa 1 – 2 – 3 – 4 – 5 – 6 – 7 Quotidiana

**QUESTIONARIO SUL SENSO DI PRESENZA**

**1/** Dai un valore (da 1 a 7) alla tua coscienza di essere presente nella stanza dell’esperimento durante il test.

Avevo una sensazione di “essere presente” nella stanza:

MAI 1 — 2 — 3 — 4 — 5 — 6 — 7 SEMPRE

**2/** Durante l’esperimento ti sei sentito più “spettatore” o “attore”?

Durante l’esperimento mi sono sentito:

SETTATORE 1 — 2 — 3 — 4 — 5 — 6 — 7 ATTORE

**3/** Quando ripensi alla stanza dove è avvenuto il test, consideri il ricordo della stanza come un’immagine che hai visto o come un posto che hai visitato? La sala mi sembrava come:

IMMAGINE VISTA 1 — 2 — 3 — 4 — 5 — 6 — 7 POSTO VISITATO

**4/** Durante l’esperimento, qual era la sensazione più forte, quella di essere nella stanza dell’esperimento o quella di essere altrove? Avevo la sensazione:

DI ESSERE ALTROVE 1 — 2 — 3 — 4 — 5 — 6 — 7 DI ESSERE NELLA STANZA DELL’ESPERIMENTO

**5/** Quanto giudichi ricco di dettagli il ricordo della stanza? Il ricordo della stanza è:

SCARSO DI DETTAGLI 1 — 2 — 3 — 4 — 5 — 6 — 7 RICCO DI DETTAGLI

**6/** Durante l’esperimento, ti sei domandato se sei realmente nella stanza dell’esperimento? Mi sono domandato di essere nella stanza dell’esperimento:

MAI 1 — 2 — 3 — 4 — 5 — 6 — 7 SEMPRE

**7/** Per ciò che riguarda il ricordo dell’esperimento, saresti capace di ricostruire correttamente la posizione del tuo corpo e le sue sensazioni? Sono capace di ricostruire la posizione del corpo e le mie sensazioni

PER NULLA 1 — 2 — 3 — 4 — 5 — 6 — 7 ASSOLUTAMENTE Sì

**8/** Durante l’esperimento, hai avuto spesso la sensazione di non comprendere ciò che accadeva attorno a te? Durante l’esperimento ho avuto la sensazione di non capire ciò che succedeva attorno a me:

MONTO SPESSO 1 — 2 — 3 — 4 — 5 — 6 — 7 MAI

**9/** Durante l’esperimento, è capitato spesso di avere l’impressione di prendere parte dell’azione che stava accadendo di fronte ai tuo occhi? Mi sono sentito parte dell’azione che stava accadendo:

MAI 1 — 2 — 3 — 4 — 5 — 6 — 7 MOLTO SPESSO

**10/** Hai avuto difficoltà a seguire ciò che accadeva durante l’esperimento? Ho avuto difficoltà:

MOLTO SPESSO 1 — 2 — 3 — 4 — 5 — 6 — 7 MAI

**QUESTIONARIO ENTATIVITA’**

**1.** Quanto hai sentito il binomio come un “tutto”, un’entità singola? Ho sentito il binomio come un’entità singola:

PER NULLA 1 – 2 – 3 – 4 – 5 – 6 – 7 – 8 – 9 COMPLETAMENTE

**2.** Pensi di aver creato un qualche tipo di legame con l’altra persona? Ho creato un legame:

PER NULLA 1 – 2 – 3 – 4 – 5 – 6 – 7 – 8 – 9 DECISAMENTE Sì

**3.** Qual è stato il grado di comunicazione con l’altra persona? Per comunicazione intendiamo qualsiasi tipo di interazione (verbale, visiva, corporea, emotiva…). La comunicazione con l’altra persona è stata:

INESISTENTE 1 – 2 – 3 – 4 – 5 – 6 – 7 – 8 – 9 PERFETTA

**4.** Hai avuto l’impressione di comprendere come pensava o agiva l’altra persona? Ho avuto l’impressione di comprendere il comportamento dell’altra persona:

PER NULLA 1 – 2 – 3 – 4 – 5 – 6 – 7 – 8 – 9 COMPLETAMENTE

**5.** Quanto pensi di esser stato/a influenzato/a dal comportamento dell’altra persona? Il comportamento dell’altra persona mi ha influenzato:

PER NULLA 1 – 2 – 3 – 4 – 5 – 6 – 7 – 8 – 9 COMPLETAMENTE

**6.** Quante cose pensi di avere in comune con l’altra persona? Le cose in comune con l’altra persona sono:

NESSUNA 1 – 2 – 3 – 4 – 5 – 6 – 7 – 8 – 9 MOLTISSIME

**7.** Quanto pensi di conoscere l’altra persona? Conosco l’altra persona:

PER NULLA 1 – 2 – 3 – 4 – 5 – 6 – 7 – 8 – 9 MOLTISSIMO

**8.** Per te, il binomio era organizzato? Ho sentito il binomio organizzato:

PER NULLA 1 – 2 – 3 – 4 – 5 – 6 – 7 – 8 – 9 COMPLETAMENTE

**9.** Quanto hai sentito motivato te e l’altra persona del binomio? Ho sentito motivati me e l’altra persona:

MOLTISSIMO 1 – 2 – 3 – 4 – 5 – 6 – 7 – 8 – 9 PER NULLA

**10.** Secondo te, sarebbe semplice per una nuova persona integrarsi al vostro binomio? Penso che sarebbe facile per una nuova persona integrarsi al binomio:

PER NULLA 1 – 2 – 3 – 4 – 5 – 6 – 7 – 8 – 9 MOLTISSIMO

**11.** Per te, il binomio si è strutturato spontaneamente? Ho sentito il binomio strutturato spontaneamente:

COMPLETAMENTE 1 – 2 – 3 – 4 – 5 – 6 – 7 – 8 – 9 PER NULLA

**12.** A che punto pensi che l’altra persona si è legata a te? L’altra persona si è legata a me:

PER NULLA 1 – 2 – 3 – 4 – 5 – 6 – 7 – 8 – 9 COMPLETAMENTE

**13.** Quanto giudichi il coinvolgimento tuo e dell’altra persona durante lo svolgimento del compito? Giudico il coinvolgimento:

SCARSO 1 – 2 – 3 – 4 – 5 – 6 – 7 – 8 – 9 ELEVATO

**14.** Secondo te, tra voi due, c’era qualcuno che aveva più controllo sull’altro e sul comportamento del binomio? Penso che c’era uno dei membri del binomio che aveva un controllo maggiore:

PER NULLA 1 – 2 – 3 – 4 – 5 – 6 – 7 – 8 – 9 COMPLETAMENTE

**15.** Hai avuto la sensazione di essere sulla stessa lunghezza d’onda dell’altra persona durante il test?
 Ho avuto la sensazione di essere sulla stessa lunghezza d’onda:

PER NULLA 1 – 2 – 3 – 4 – 5 – 6 – 7 – 8 – 9 COMPLETAMENTE

**16.** Hai avuto la sensazione che l’altra persona capiva come tu pensavi durante il test? Ho avuto la sensazione che l’altra persona capiva come pensavo:

PER NULLA 1 – 2 – 3 – 4 – 5 – 6 – 7 – 8 – 9 COMPLETAMENTE
